# Synthesis of qualitative evidence on community experiences and perceptions of *Plasmodium knowlesi* malaria and factors influencing prevention and healthcare-seeking behaviours in Malaysia

**DOI:** 10.1186/s12936-026-05785-4

**Published:** 2026-01-12

**Authors:** Mila Nu Nu Htay, Nik Hamzah Nik Mohd, Mon Mon Thawda Oo, Lin Phyo Phyo San, Adinegara Lutfi Abas

**Affiliations:** 1 Centre for Population Health (CePH), Department of Social and Preventive Medicine, Faculty of Medicine, Universiti Malaya, Kuala Lumpur, Malaysia; 2 Department of Community Medicine, Faculty of Medicine, Manipal University College Malaysia, Melaka, Malaysia; 3 Faculty of Medicine, Lincoln University College, Petaling Jaya, Malaysia; 4 Faculty of Medicine, Manipal University College Malaysia, Melaka, Malaysia; 5 Department of Medicine, M. Kandiah Faculty of Medicine & Health Sciences, Universiti Tunku Abdul Rahman, Sungai Long, Selangor Malaysia

**Keywords:** Malaria, *Plasmodium knowlesi*, Malaysia, Prevention, Health-seeking behaviours

## Abstract

**Background:**

Malaria remains a significant public health challenge, and *Plasmodium knowlesi* malaria has become the predominant cause of malaria in Malaysia. Despite progress in eliminating nonzoonotic malaria species, Malaysia continues to face challenges in controlling *P. knowlesi*. While epidemiological and vector control studies are reported, less is known about the community-level sociocultural dynamics influencing prevention behaviours. Therefore, this qualitative evidence synthesis (QES) aims to consolidate existing evidence on community experiences and perceptions related to *P. knowlesi* malaria, as well as the social, cultural, and contextual factors influencing prevention and healthcare-seeking behaviours in Malaysia.

**Methods:**

This QES protocol has been registered in PROSPERO (CRD 420251045457). A systematic literature search was conducted in electronic databases, and data analysis followed Thomas and Harden’s thematic synthesis method.

**Results:**

The QES included five qualitative and mixed-method studies published between 2022 and 2024 that explored community perspectives on *P. knowlesi* malaria in Malaysia. Three analytical themes were synthesized: (1) ‘Knowledge and Lived Realities Shape Community Risk Perception of Knowlesi Malaria’, suggesting community understanding of *P. knowlesi*, fear of hospitalization and income loss influencing health-seeking behaviours; (2) ‘Environmental, Structural, and Social Barriers Constrain Community Engagement with Malaria Prevention and Healthcare Seeking’, where environmental exposure, occupational risks, challenges to the use of personal protection, and access barriers were major determinants; and (3) ‘Malaria Prevention Practices Reflect Local Knowledge, and Availability of Formal Prevention Measures’, highlighting the application of natural and household remedies for prevention, while using formal preventive measures.

**Conclusions:**

This QES consolidates the available evidence for *P. knowlesi* malaria control strategies including prevention and healthcare seeking. It highlights that malaria prevention behaviours are shaped not only by knowledge on transmission and diseases, but also by the social, environmental, and cultural realities in local context. Therefore, integrating local community perspectives and challenges into prevention and vector control programs could enhance the sustainability and equity in rural areas in Malaysia.

**Supplementary Information:**

The online version contains supplementary material available at 10.1186/s12936-026-05785-4.

## Introduction

Malaria remains a significant public health challenge, a rise in global incidence between 2019 and 2021 [1]. The emerging zoonotic form, *Plasmodium knowlesi* malaria, has gained prominence in Southeast Asia [2]. *P. Knowlesi* malaria cases have been reported in Malaysia, Indonesia, Thailand, and Cambodia [3–6], with environmental mapping indicating the highest occurrence in Malaysia and Indonesia [3]. The transmission of *P. knowlesi* is related to complex ecological dynamics among humans, the primary reservoir, macaque monkeys and mosquito vectors [7]. Therefore, the emergence of *P. knowlesi* infection is a challenge for malaria elimination efforts.

**Fig. 1:**
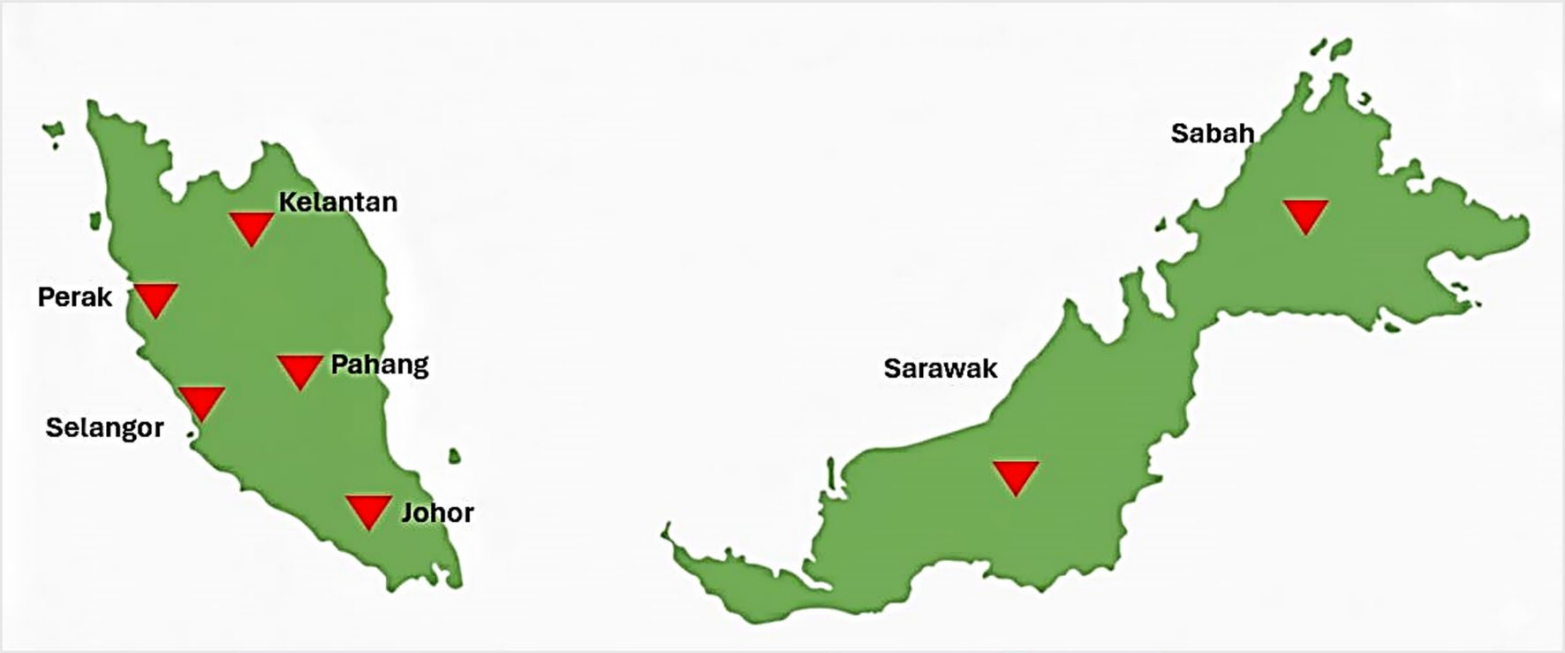
Distribution of *Plasmodium knowlesi* malaria cases in Malaysia (2011–2018), highlighting Kelantan, Pahang, Perak, Selangor, Johor [11], and Sabah, Sarawak states [12] with the highest reported cases or key epidemiological relevance

Malaysia comprises Peninsular Malaysia and Borneo Island. Peninsular Malaysia consists of 11 states and two federal territories. While Sabah and Sarawak states are located in Borneo Island. *P. knowlesi* imposes a unique challenge in prevention and control activities; emerged and mainly occurring in Sabah and Sarawak [9]. In Peninsular Malaysia, a spatial and temporal analysis reported that Kelantan (29.35%), Pahang (24.07%), Perak (18.94%), Selangor (7.88%), and Johor (6.40%) had the highest number of knowlesi malaria cases between 2011 and 2018 [8] (Fig. 1). Conventional control measures, such as insecticide-treated nets and indoor residual spraying, are intended to prevent human‒vector‒human transmission. However, they have limited effectiveness for individuals involved in outdoor activities and farming settings [10].

The population at risk of *P. knowlesi* malaria is mainly in Borneo [11] and some Orang Asli settlements in Peninsular Malaysia [12]. The Orang Asli, the indigenous peoples, lives predominantly in non-urban areas. They rely on forest resources and livelihoods [13], which increases their exposure to malaria vectors and zoonotic infections such as *P. knowlesi*. The people who spent overnight in the forest did not use mosquito repellents, and a previous history of malaria infection was found to be associated with *P. knowlesi* malaria infection [11]. Complex environmental factors, population behaviours, misconceptions about disease, cultural and traditional beliefs, and health-seeking behaviours influence disease occurrence and treatment-seeking behaviours [10, 14]. These ecological and occupational exposures, combined with traditional beliefs and limited healthcare access, shape how rural populations perceive and respond to malaria risk.

While the epidemiology, clinical features, and ecological aspects of *P. knowlesi* have been widely studied [15–17], there remains limited understanding of the social and cultural contexts influencing prevention and healthcare-seeking behaviours. Public health interventions are more successful if they are planned and organized to be relevant to cultural factors while taking into consideration the contextual situation [18]. Qualitative evidence provides in-depth contextual insights that complement quantitative surveillance data by uncovering beliefs, behaviours, and cultural factors that influence malaria prevention and healthcare-seeking. A study conducted in India revealed that health seeking malaria treatment is influenced by trust in healthcare providers and their beliefs in traditional medicine and healers [19].

However, comprehensive qualitative evidence exploring people’s experiences, perceptions, and health-seeking behaviour related to *P. knowlesi* malaria in Malaysia is still limited. An in-depth understanding of perceptions of *P. knowlesi* malaria, beliefs about this illness, prevention measures, and healthcare-seeking behaviours is crucial for informing culturally appropriate and effective interventions in Malaysia. Therefore, this qualitative evidence synthesis (QES) was performed to explore community experiences; perceptions related to *P. knowlesi* malaria; and social, cultural, and contextual factors that influence prevention strategies and healthcare-seeking behaviour in Malaysia.

## Methods

This qualitative evidence synthesis protocol has been registered in PROSPERO (CRD 420251045457).

### Inclusion criteria

Primary qualitative research or mixed-method research was included in this evidence synthesis. Studies which explored and/ or included community experiences, perceptions, beliefs, attitudes, and knowledge related to *P. knowlesi* malaria in Malaysia (including phenomenology, ethnography, case studies, etc.) and published in English were included.

### Exclusion criteria

Studies related to healthcare providers, policy makers, and biological, clinical, genetic, and pharmacologically related quantitative studies about *P. knowlesi* were excluded. Details of the inclusion and exclusion criteria are provided in Table 1.

**Table 1:** Inclusion and exclusion criteria for study selection

Inclusion	Exclusion
Study population: community members, community leaders, residents, or populations living in Malaysia	Study population: Studies focused solely on healthcare providers without including community members
Studies explored and/ or included experiences, perceptions, beliefs, attitudes, and knowledge related to *Plasmodium knowlesi* malaria	Studies focused only on the biological and genetic perspective of *Plasmodium knowlesi* infection, treatment of patients with *P. knowlesi* malaria, diagnosis, and pharmacological perspectives
Qualitative studies or Mixed-method studies	Quantitative studies (e.g., cross-sectional studies, experimental studies, other quantitative primary research studies, systematic reviews, scoping reviews, commentaries, conference proceedings, editorials, and opinions)
Studies conducted within Malaysia, focusing on any state or region	Studies conducted in other countries
Studies published in English language	

### Search strategy

We attempted to search databases, including Medline, Embase, PsycInfo, the Cochrane Library, Web of Science, and CINAHL, to identify all relevant studies. The database search date ranged from the commencement of the database until May 2025 to identify the articles relevant to *P. knowlesi* malaria. Searches were completed on 4 May 2025. The forward citations of the included studies were searched in Google Scholar manually to track the current literature. Grey literature was searched using the relevant OpenGrey database. An extensive literature search was carried out to capture the comprehensive synthesis of the qualitative evidence.

Search terms were developed by applying the SPIDER framework [20] to capture a sample, phenomenon of interest, design, evaluation, and type of research. The complete search strategy for each database, including keywords and Boolean operators, is detailed in Appendix Table 1 to ensure replicability.

### Selection of studies

We imported all the search results into Rayyan, a web-based tool that facilitates screening and selection of studies for systematic reviews [21]. Two independent researchers (MNNH and LPPS) conducted independent study selection processes and followed the Preferred Reporting Items for Systematic Reviews and Meta-Analyses (PRISMA) guidelines. Two independent reviewers (MNNH and LPPS) screened the titles and abstracts. After that, screening of the full-text articles was conducted. Any disagreements were resolved through discussion between researchers, who consulted with a third researcher (MMTO). We recorded the reasons for the exclusion of full-text studies. Data from the final set of included studies were reviewed and extracted.

### Data extraction

Data extraction was carried out as two parts, (1) study information and demographic data extraction, and (2) qualitative data extraction. Reviewers (MNNH, NHNM) extracted study information and demographic data from the included studies. Data extraction was conducted to capture details such as the author's name, publication year, journal, record number, study characteristics (including methodology, method, phenomena of interest, study setting, geographic location, cultural context, participant characteristics, and data analysis methods).

Qualitative data (themes, subthemes, participants quotations) regarding perceptions, experiences, and prevention or healthcare-seeking behaviours were extracted from the included studies. Only the qualitative components of mixed-method studies were included for data extraction and synthesis. A review author (MMTO) independently checked and verified the accuracy and appropriateness of the data extraction.

### Quality assessment

The methodology quality assessment of the included articles was conducted via the Cochrane Qualitative Methodological Limitations Tool (CAMELOT) [22]. We did not exclude studies on the basis of the assessment results of methodological limitations. We applied this information in the assessment of the confidence level for each theme of the review findings.

### Data analysis

The extracted data were imported into and analysed via NVivo software (version 15). A thematic evidence synthesis approach was applied for evidence synthesis following Thomas and Harden’s (2008) three-step process [23]. The review authors applied the following steps in the data analysis process. (1) Familiarizing with the extracted qualitative data and initial coding: two review authors read the included articles to familiarize them with and immerse them in the data. Two independent reviewers generated the initial codes inductively. Discussion and consensus were reached after the initial coding steps. (2) Development of descriptive subthemes and themes: the review authors grouped the codes as subthemes according to their relationships, similarities, and differences. The authors' team reviewed overall subthemes and themes for coherence, separation or merging of the developed themes. (3) Generation of analytical themes: the authors' team reviewed and discussed the findings to generate an analytical theme that was interpreted beyond the original findings to contribute to future applications.

## Results

### Search results

**Fig. 2:**
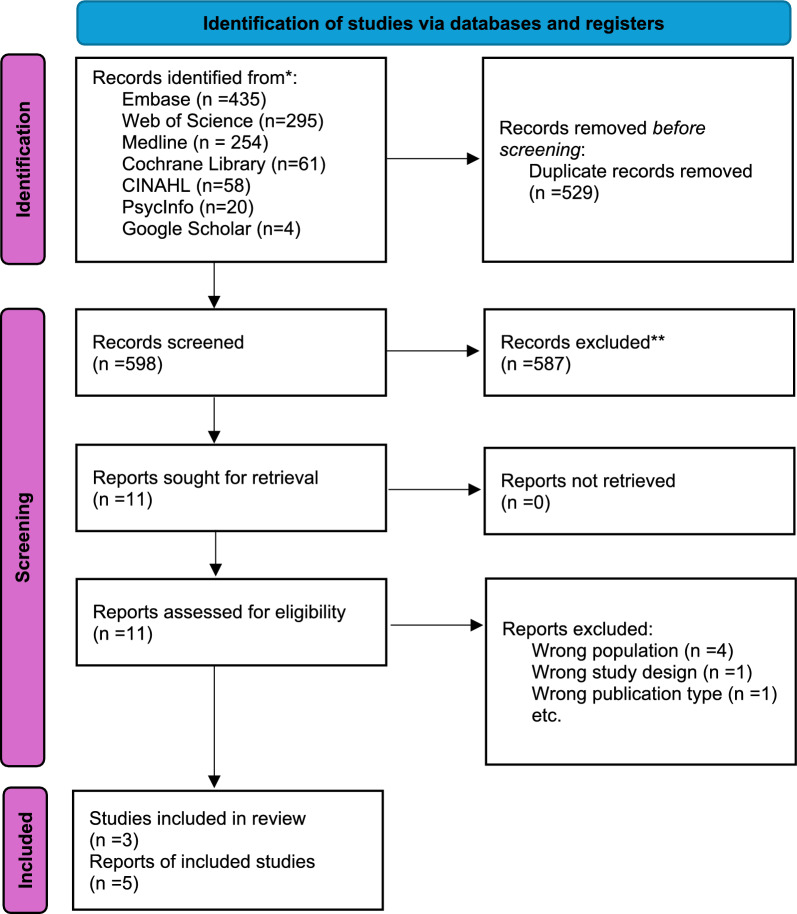
PRISMA flow diagram of study selection

A total of records 1127 were identified through database searches. After removing duplicates, the remaining records (n = 598) were screened by title and abstract, followed by full-text review. Studies that did not meet the inclusion criteria were excluded. Finally, a total of five studies were included in our evidence synthesis (Fig. 2). Among them, four studies were qualitative [24–27], and one study was a mixed-method study [28].

### Study settings and sample population

The characteristics of the included studies are presented in Appendix Table 2. Three studies were conducted in Sabah [24–26], one study was conducted across the central spine forest range in Peninsular Malaysia [28], and one study was conducted in Johor, Pahang, and Kelantan states in Malaysia [27]. The sample population included villagers in a rural community (villagers), community leaders, indigenous people, and military personnel.

### Quality assessment

The methodological quality assessments were conducted according to the CAMELOT tool; three studies had minimal concern [24–26], and two studies were categorized as having minor concern [27, 28]. Some methodological limitations such as unclear theoretical background, concepts, researchers’ consideration of equity, diversity, and inclusion in the study process, and limited reflexibility might imposed to potential bias in interpretation. The details of the methodical quality assessment are presented in Appendix Table 3.

### Confidence in findings

The study findings were evaluated for methodological limitations, coherence, adequacy, and relevance via the confidence in the evidence from reviews of qualitative research (CERQual) approach. Two review authors assessed the components of the assessment and determined the overall confidence of the review findings. Methodological limitations were assessed based on the quality and rigor of the included qualitative and mixed-methods studies. When methodological concerns were identified in the primary studies supporting a theme, the confidence level for that theme was considered and graded accordingly. This assessment informed the overall confidence in the synthesized themes. High confidence themes were supported by studies with minor methodological limitations, consistent and coherent findings, and relevant data. Meanwhile, themes rated as moderate confidence if there were minor to moderate concerns in one or more domains, such as limited data adequacy or moderate methodological limitations in the contributing studies.

Among the nine subthemes, three were assessed as having high confidence, whereas the remaining six were rated with moderate confidence according to the GRADE-CERQual assessment. The details of the assessment are presented in Table 2.

**Table 2:** GRADE-CerQual Summary of Qualitative Evidence Synthesis Results

	Findings	Methodological limitations	Coherence	Adequacy	Relevance	Overall confidence	Contributing records
Theme 1. Knowledge and lived realities shape community risk perception of knowlesi malaria							
1	Sub-theme 1.1. “Awareness of malaria and vector" Participants from the states with high numbers of malaria cases aware about malaria parasite, monkey reservoirs, and mosquito vector. However, in Peninsular Malaysia, awareness still needs to be improved.	No/very minor concerns	Minor concerns	Moderate concerns	No/very minor concerns	Moderate confidence	Kader Maideen et al. 2022; Naserrudin et al. 2023
2	Sub-theme 1.2. “Perceived Severity and Risk" The complexity of perceived severity and risk perception was reported in the studies. Some participants were worried about getting malaria and admission to the hospital. Self-motivation is a critical factor influencing individual and household-level preventive behaviors against *Plasmodium knowlesi* malaria. The participants demonstrated their self-motivation and took responsibility for personal protection measures. In contrast, some people considered themselves lucky and may not get malaria.	No/very minor concerns	No/very minor concerns	Minor concerns	No/very minor concerns	Moderate confidence	Kader Maideen et al. 2022; Naserrudin et al. 2023
Theme 2. Environmental, structural, and social barriers constrain community engagement with malaria prevention and healthcare seeking							
3	Sub-theme 2.1. “Ecological and Environmental Challenges in Malaria Prevention" Participants raised the concern of deforestation, human settlement, and an increase in human-wildlife interaction, particularly with monkeys. These ecological interactions not only lead to crop damage and economic loss but also intensify the risk of zoonotic malaria transmission.	No/very minor concerns	No/very minor concerns	Moderate concerns	No/very minor concerns	Moderate confidence	Naserrudin et al. 2023; Naserrudin et al. 2023; Naserrudin et al. 2023
4	Sub-theme 2.2. “Structural and Access Barriers " Structural and transport barriers are reported by some participants living in rural or remote areas to seek healthcare or to provide prevention programmes. Limited infrastructure, such as inconsistent electricity supply, internet connectivity, and unreliable water systems, which affect daily living and diminish the feasibility of adopting protective practices and increasing exposure to mosquito vectors.	No/very minor concerns	No/very minor concerns	Minor concerns	No/very minor concerns	High confidence	Azlan et al. 2023; Naserrudin et al. 2023; Naserrudin et al. 2023; Naserrudin et al. 2023
5	Sub-theme 2.3. “Social and Household Barriers" Communities in rural areas faced social and household-level barriers. Family responsibilities, such as caring for children, deter individuals from seeking treatment. Participants consistently highlight how daily livelihood and activities in rural communities, increased the exposure to mosquito vector.	No/very minor concerns	No/very minor concerns	Moderate concerns	No/very minor concerns	Moderate confidence	Naserrudin et al. 2023; Naserrudin et al. 2023; Naserrudin et al. 2023
6	Sub-theme 2.4. “Doubts on Effectiveness of Preventive Measures and Practical Limitations" Some community people expressed doubts on effectiveness towards the formal preventive measures such as protective clothing, mosquito repellents, or bed nets. While some those who took malaria prophylaxis reported the doubt on effectiveness.	No/very minor concerns	No/very minor concerns	Minor concerns	No/very minor concerns	High confidence	Azlan et al. 2023; Naserrudin et al. 2023; Naserrudin et al. 2023
Theme 3. Malaria prevention practices reflect local knowledge, and availability of formal prevention measures							
7	Sub-theme 3.1. “Natural and Household Remedies" In rural communities, natural preventive remedies are commonly used to avoid mosquito bites. These practices are driven by accessibility, familiarity, and the perceived effectiveness of strong smells and smoke to repel mosquitoes. In addition, manual techniques to ward off or kill mosquitoes are mentioned by some participants.	No/very minor concerns	No/very minor concerns	Moderate concerns	No/very minor concerns	Moderate confidence	Naserrudin et al. 2023; Naserrudin et al. 2023
8	Sub-theme 3.2. “Modern and Formal Prevention Tools" The participants are generally recognized for the effectiveness of formal prevention tools. While in Peninsular Malaysia, prophylactic medications for malaria are valued, especially by those who have previously experienced febrile illnesses, with personal experiences influencing adherence. Bed nets, particularly those provided by the health department, are accepted for nighttime protection.	No/very minor concerns	No/very minor concerns	Minor concerns	No/very minor concerns	High confidence	Azlan et al. 2023; Naserrudin et al. 2023; Naserrudin et al. 2023
9	Sub-theme 3.3. “Tailored prevention measures for local context are needed" There is a need for more effective, affordable, and acceptable prevention tools, including improved bed nets and repellents. These insights reflect a gap between the design of formal prevention measures and the realities of use in forested or rural settings, where electricity and fans may not be available.	No/very minor concerns	No/very minor concerns	Moderate concerns	No/very minor concerns	Moderate confidence	Naserrudin et al. 2023; Naserrudin et al. 2023

### Synthesis findings

#### Theme 1. Knowledge and lived realities shape community risk perception of knowlesi malaria

Community knowledge of Knowlesi malaria varies according to geographic location.

##### Subtheme 1.1. Awareness of malaria and vectors

Participants from states with high numbers of malaria cases were aware of malaria parasites, monkey reservoirs, and mosquito vectors [25]. However, in Peninsular Malaysia, awareness still needed to be improved, as a participant said, “I’m not familiar with monkey malaria. I have never heard of it” [28].

In rural areas of Sabah, the participants reported their experiences of participation in malaria-related research [26]. This might contribute to their awareness of parasites and vectors. While some respondents had a positive perception of contributing to research as they could learn from others, “I learn about malaria; I learn about how other people avoid the mosquitoes… I did not go to secondary school… I appreciate all this new knowledge” [25]. Therefore, participatory action research might be beneficial for promoting awareness and sharing knowledge within rural communities.

##### Subtheme 1.2. Perceived severity and risk

The complexity of perceived severity and risk perception was reported by the studies. Some participants were worried about contracting malaria and being admitted to the hospital [26]. This fear was rooted in socioeconomic constraints and family commitment, which might be affected by sickness [26, 28]. Their concern for the risk of malaria was also extended to their children [26].

Self-motivation was a critical factor influencing individual- and household-level preventive behaviours against *P. knowlesi* malaria. The participants revealed their self-motivation and took responsibility for personal protection measures [26, 28]. This internal motivation was driven by the perceived impact of disease, as mentioned by a participant:

“we need to try our best to avoid contact with these mosquitoes […] Moreover, I do not want to be admitted to the hospital. I do not want to get sick” [26]. This internal drive was translated into proactive behaviours and consistent actions to prevent mosquito bites.

In contrast, some people considered themselves lucky and might not develop malaria [26]. However, some people believed that they were resistant to Knowlesi malaria infection. Therefore, further enhancement of malaria health literacy is needed, especially in areas with high numbers of malaria cases.

These insights reflected a complex interplay of knowledge, beliefs, and lived realities that influenced how communities understood and responded to the threat of *P. knowlesi* malaria.

#### Theme 2. Environmental, structural, and social barriers constrain community engagement with malaria prevention and healthcare seeking

In this evidence synthesis, multifaceted barriers that hinder community efforts to prevent malaria and seek healthcare in a timely manner were identified [24, 26, 27], especially in the rural areas of Borneo [24, 26].

##### Subtheme 2.1. Ecological and environmental challenges in malaria prevention

The participants raised concerns about deforestation, human settlement, and increased human‒wildlife interactions, particularly with monkeys [24]. These ecological interactions not only led to crop damage and economic loss but also increased the risk of zoonotic malaria transmission, such as *P. knowlesi* malaria [24–26]. A participant revealed the concern as follows;

“The monkeys, they disturb our farm, our plants, fruit trees… we hope that someone could find a way.” [25].

##### Subtheme 2.2. Structural and access barriers

Structural and transport barriers were reported by participants living in rural areas seeking healthcare [24]. Moreover, some delays in the distribution of protective tools, such as bed nets, had been reported [24].

Limited infrastructure, such as inconsistent electricity supplies, internet connectivity, and unreliable water systems, disrupted daily living. In addition, these diminished the feasibility of adopting protective practices, and increased exposure to mosquito vectors [24, 26]. Insufficient electricity availability in rural Sabah led to more exposure to mosquitoes in darkness [24], which were unable to sleep under the bed net because of a lack of airflow [26].

Access to the water supply was crucial in villages, where they had limited access to a pipe water supply [24, 26]. When villagers entered the forest to access water, their exposure to mosquito vectors increased, increasing the risk of malaria infection, as explained by one participant: “despite knowing the risk of mosquito bites and malaria […] they still need to walk and hike into the forest. That is where the water supply comes from, the gravity water.” [26]. Notably, these barriers were primarily reported by participants in rural areas of Sabah [24, 26], whereas they were not mentioned by participants from Peninsular Malaysia [27, 28].

##### Subtheme 2.3. Social and household barriers

Communities in rural areas faced social and household-level barriers. Family responsibilities, such as caring for children, deterred individuals from seeking treatment: “So people will stay home and not go to the clinic” [24, 26].

Tradition and housing conditions, such as having open gaps and a lack of mosquito-proofing, further exacerbated exposure to vectors [26]. The participants consistently highlighted how daily livelihoods and activities in rural communities, such as farming, rubber tapping, fishing, hunting, and tending to livestock, increased exposure to mosquito vectors [24–26]. “It is not easy to avoid malaria in this village. People need to go out to search for vegetables, food, and their living” [24]. Even children participated in high-risk activities such as fishing and farming, further increasing community vulnerability [26].

Environmental sanitation was essential for the effective prevention and control of mosquito breeding. However, some rural communities identified poor waste management, improper disposal of containers, and a lack of routine cleaning as key contributors to the proliferation of mosquito breeding sites [24, 25].

Additionally, cultural and recreational practices such as evening outdoor social gatherings (e.g., aramaiti) increased the risk of mosquito bites [26]. These everyday social and cultural practices within rural settings contributed to community vulnerability to *P. knowlesi* malaria. Therefore, future interventions should take cultural perspectives into account and incorporate them into malaria prevention and control strategies.

The participants (villagers) highlighted the ecological changes, particularly deforestation, inadequate waste management, and seasonal water stagnation, that had created breeding grounds for malaria vectors [24–26].

##### Subtheme 2.4. Doubts on the effectiveness of preventive measures and practical limitations

Some community people expressed doubts about the effectiveness of formal preventive measures of malaria such as protective clothing, mosquito repellents, or bed nets [24, 26, 27]. Furthermore, the nature of the workplace might not be suitable for some protective tools [26]. Others find such tools inconvenient or unnecessary, particularly when engaging in work or outdoor activities such as farming, fishing, or hunting [24].

While some people reported doubts about the effectiveness of malaria prophylaxis, “We can’t be sure that it has an effect because even when we consume (prophylaxis), we could still get mosquitoes bites. So for prophylaxis, it is more for internal precaution.” [27]. Some participants disclosed their neglected preventive measures [26, 27].

Economic and health-related factors limited the consistent use of formal malaria prevention tools within the community. Despite recognizing the potential benefits, their regular use was hindered by financial constraints [27]. Additionally, concerns about health risks, particularly those related to the smoke emitted by mosquito coils, further discourage its use. One participant expressed concern about the negative side effects on children, such as respiratory irritation or runny noses [27]. The complex nature of these barriers suggested that effective malaria prevention strategies must align with the community’s livelihood patterns and environmental context.

#### Theme 3. Malaria prevention practices reflect local knowledge and availability of formal prevention measures

The community's malaria prevention practices reflected local ecological knowledge, cultural traditions, and the accessibility of formal prevention tools such as mosquito repellents and bed nets.

##### Subtheme 3.1. Natural and household remedies

In rural communities, natural preventive remedies were commonly used to prevent mosquito bites [25, 26]. Locally available materials such as garlic, aromatic plants, and coconut fibre smoke were protective measures against mosquito bites [25]. Various natural plants, including *Lantana camara*, lemongrass, *Cymbopogon nardus*, Thai basil, *Ocimum basilicum*, and local wild edible ginger, were also applied as natural remedies [25]. Burning coconut fibres to release smoke was also a natural measure to avoid mosquito bites [25]. These practices were driven by accessibility, familiarity, and the perceived effectiveness of strong smells and smoke to repel mosquitoes. In addition, manual techniques to ward off or kill mosquitoes were mentioned by some participants [25, 26].

##### Subtheme 3.2. Modern and formal prevention tools

The participants were generally recognized for the effectiveness of formal prevention tools [25, 27]: “For me, the most effective would be mosquito coil and bed net because from my own experience, they can reduce the chances of getting bitten by mosquitoes” [27]. Mosquito coils were commonly used indoors but were perceived as less effective for outdoor activities because of their limited smoke coverage and short duration of effect [25]. Similarly, mosquito repellents were preferred for their portability and convenience during forest activities and outdoor environments [27].

In Peninsular Malaysia, general malaria preventive measures, including prophylactic medication, were valued particularly by individuals who had previously experienced febrile illnesses, as their personal experiences influencing adherence: “I had previous experience not taking (prophylaxis) because I was too preoccupied with other tasks at the time… and coincidentally I had an on-and-off fever (while in the forest). That experience (although not knowlesi malaria related) made me feel like it is not worth risking it. So since then, I made sure that I take the medicine (prophylaxis).” [27]. Bed nets, particularly those provided by health departments, were widely used for nighttime protection [25, 27].

##### Subtheme 3.3. Tailored prevention measures for the local context are needed

There was a need for more effective, affordable, and acceptable prevention tools, including improved bed nets and repellents [24, 26]. “The mosquitoes can fly through the bed net holes. The holes are relatively large (while pointing to the blue nets). The bed nets, too, it is hot. When I sleep under it, it is hot” [26]. One participant expressed a hopeful wish for the advancement of malaria vaccine distribution in Malaysia: “I believe the most effective way could be by vaccination. Why can they design the COVID-19 vaccine rapidly but not for this disease (malaria)?” [24] These insights reflected a gap between the design of formal prevention measures and the realities of their use in forested or rural settings, where electricity and fans might not be available. The details of the codes, subthemes, and themes are presented in Appendix Table 4.

**Fig. 3:**
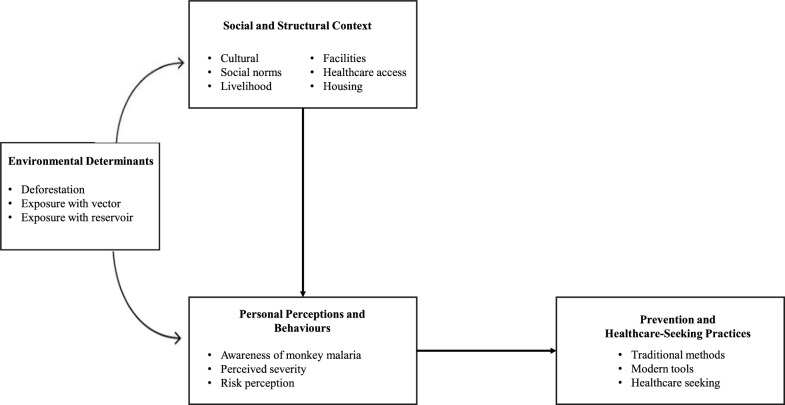
Conceptual Framework of Factors Influencing Prevention and Healthcare-Seeking Behaviours in Malaysia

The evidence synthesis findings and concepts are presented in Fig. 3.

## Discussion

### Summary of key findings

Based on the five included studies, the community's perception and experience of knowlesi malaria in Malaysia is shaped by a complex interplay of knowledge, environmental challenges, and socio-structural barriers. We found that knowledge about *P. knowlesi* malaria varies across geographic regions, with lower awareness reported in Peninsular Malaysia compared to high-incidence areas of Sabah. This finding reflects the influence of the malaria infection rate, local research activities, and community awareness programs. Although a study conducted in Kelantan, Peninsular Malaysia reported acceptable knowledge and awareness about general malaria infection [29], there is still a need for region-specific health education initiatives in Peninsular Malaysia.

This pattern mirrors findings from Thailand and Vietnam, where community malaria literacy also varies geographically and correlates with exposure risk and health outreach intensity [4, 5]. In Indonesia, forest-dependent and migrant populations demonstrate similar variations in zoonotic malaria awareness, reinforcing the need for locally tailored communication strategies across Southeast Asia [30].

Perceived severity and risk appear to be influenced by biomedical knowledge and realities in a socioeconomic context. Fear of hospitalization due to knowlesi malaria, loss of income, and family commitment may contribute to the fear of contracting malaria infection [26, 28]. These perceived severity and barriers to seeking health during illness further motivate some villagers to adopt preventive behaviours. This finding aligns with health belief models, which highlight perceived susceptibility and severity towards the adoption of prevention measures [31].

Environmental and structural factors, particularly in rural Borneo, pose significant constraints. Ecoepidemiological changes, such as deforestation, wildlife encroachment, and increasing proximity to macaque habitats, are reported to lead to a higher frequency of mosquito bites [7, 14, 24–26]. Such accounts provide support for existing theories that anthropogenic activities are associated with increased *P. knowlesi* transmission. This mirrors observations in other zoonotic malaria-endemic areas like the Greater Mekong Subregion (GMS) and Indonesia, where land-use changes and forest encroachment have been identified as key drivers of human exposure and disease risk [30, 32].

Comparable evidence from Indonesia and Cambodia demonstrates how land-use changes and forest fragmentation heighten vector–host contact [3, 30]. While Thailand’s cross-sectoral surveillance models integrating human, animal, and environmental data exemplify regional approaches to managing zoonotic transmission [4, 32]. This expanded comparison highlights that Malaysia’s challenges are part of a broader Southeast Asian eco-social dynamic that demands regional collaboration in vector ecology, community engagement, and surveillance.

Rural communities face additional adversity from infrastructures, such as poor roads, interrupted electricity supplies and inadequate water resources [24, 26]. Similar structural challenges, including distance to health facilities and infrastructure limitations, have been documented in the rural areas in Indonesia, hindering timely diagnosis and treatment for malaria patients [33]. It highlights the importance of developing strategies including community perspectives, improving infrastructure, and incorporate behavioural interventions to control zoonotic malaria in Southeast Asia countries [32, 33].

In terms of social responsibilities, particularly for women and care givers, making a journey to a health facility could be a challenge due to the time taken, childcare responsibilities, and the cost of travel [24–26]. Additionally, concerns about how effective and affordable tools such as mosquito coils, repellents and bed nets suggest that these interventions are not always use harmoniously. These concerns align with international studies that demonstrated practicality, side effects, and financial capacity as factors in preventive behaviour [10, 19].

Traditional prevention practices (e.g., smoke, aromatic plants) remain common due to accessibility and familiarity, however the health risks remain underexplored. This highlights the need for safer, culturally acceptable alternatives that can be feasibly used in rural settings.

### Implications for policy and strategy

At a community level, these findings highlight the fact that the battle against zoonotic malaria cannot be achieved only with the use of vector control interventions when human exposure is intricately tied to location and occupational-related context. Consequently, control strategies should be appropriate for the social realities of forest-fringe communities.

Within the framework of Malaysia’s National Malaria Elimination Strategic Plan (NMESP) 2021–2030 [34], these findings indicate that sustaining zero indigenous human malaria requires explicit integration of strategies that address zoonotic *P. knowlesi* transmission. As human-only malaria species decline, zoonotic malaria presents the main barrier to elimination, and policies and strategies must adapt accordingly.

The analytical themes from this QES provide specific directions for future actions.

Theme 1 emphasizes the importance of culturally adapted health communication to improve health literacy about zoonotic transmission.

Theme 2 highlights structural barriers such as long travel distances and transportation challenges supporting the need for decentralized rural health delivery systems.

Theme 3 indicates the value of co-developing acceptable preventive tools with communities, including improved protective clothing, treated hammocks, and culturally acceptable repellents.

Taken together, these insights suggest that Malaysia’s elimination pathway transition could expand the scope from a predominantly biomedical paradigm toward a socio-ecological and community-centred approach. These insights and strategies will strengthen Malaysia’s ability to prevent reintroduction of human malaria while simultaneously mitigating rising zoonotic transmission.

### Strengths and limitations

This qualitative evidence synthesis has some limitations that should be considered when interpreting the findings. The synthesis included only five eligible studies, and which might lead to the risk of publication bias. While these studies provided valuable insights, they were conducted primarily in rural areas of Sabah and among indigenous or mixed community populations in Peninsular Malaysia. Because of this limited geographic and population coverage, it was unable to perform a sub-analysis comparing rural versus semi-urban or indigenous versus non-indigenous community perspectives. Therefore, the synthesized findings may not be generalizable to all community experiences across Malaysia. Although this QES focused on *P. knowlesi* malaria, participants in the included studies may have described their general malaria prevention practices and experiences rather than species-specific behaviours. Hence, some findings may partly reflect broader malaria prevention strategies rather than those unique to *P. knowlesi* infection. Lastly, this synthesis included only studies published in English which may have introduced language bias.

Although there are inevitable limitations, the review team conducted the quality appraisal using the CAMELOT tool and confidence assessment with the CERQual approach. In addition, independent study selection, synthesis, and consensus discussions among reviewers were used to enhance analytical rigor and minimize individual bias.

## Conclusion and recommendations

This qualitative evidence synthesis provides the consolidated qualitative evidence on community experiences and perceptions of malaria in Malaysia, offering insights to intervention and prevention strategies. The key findings highlight that while general malaria awareness exists, specific knowledge of zoonotic is limited in some regions, and communities face significant structural, environmental, and social barriers to effective prevention and timely healthcare seeking especially in rural areas.

To address these challenges, our recommendations are directly aligned with the three analytical themes.

Theme 1 (Risk Perception): Culturally sensitive and region-specific health education is necessary to mitigate the community's limited knowledge of zoonotic transmission and to enhance risk perception.

Theme 2 (Structural Barriers): Adaptive vector control strategies suitable for the rural population are required, such as protective permethrin insecticide-treated clothing, mosquito repellents, and solar-powered long-lasting insecticidal net fans. Furthermore, improving environmental sanitation and providing basic necessities such as clean water, electricity, and internet access in forest-fringe villages can further enhance lifestyle behaviours and support malaria prevention efforts.

Theme 3 (Local Practices): Interventions should be co-designed with communities by leveraging local knowledge and traditional practices.

Future work should explore layered strategies combining environmental management, mobile outreach, and community-tailored education, as well as scientific evaluations of traditional plant-based repellents for safe and sustainable use.

## Supplementary Information

Supplementary material 1.

## Data Availability

No datasets were generated or analysed during the current study.

## Abbreviations

CAMELOT: CochrAne qualitative Methodological LimitatiOns Tool
CERQual: Confidence in the Evidence from Reviews of Qualitative research
CINAHL: Cumulative Index to Nursing and Allied Health Literature
LLITN: Long-Lasting Insecticide-Treated Net
*P. knowlesi*: *Plasmodium knowlesi*
*P. falciparum*: *Plasmodium falciparum*
*P. vivax*: *Plasmodium vivax*
PRISMA: Preferred Reporting Items for Systematic reviews and Meta-Analyses
PROSPERO: International Prospective Register of Systematic Reviews
QES: Qualitative Evidence Synthesis
SPIDER: Sample, Phenomenon of Interest, Design, Evaluation, and Research Type
WHO: World Health Organization
