# Supplementary material for: Synthesis of qualitative evidence on community experiences and perceptions of Plasmodium knowlesi malaria and factors influencing prevention and healthcare-seeking behaviours in Malaysia
Source: Malar J. 2026 Jan 12;25:89. doi: 10.1186/s12936-026-05785-4 (PMC12888265; doi:10.1186/s12936-026-05785-4)
Supplement: Supplementary file 1 — Supplementary material 1. [file 12936_2026_5785_MOESM1_ESM.pdf]

Appendix

Appendix Table 1. Search terms to apply for database searches

SPIDER TOOL		Search terms
S	Sample	(“community” OR “population” OR “villagers” OR “residents” OR “rural community”) AND
PI	Phenomenon of interest	(“*Plasmodium knowlesi*” OR “knowlesi malaria” OR “malaria” OR “zoonotic malaria”) AND
D	Design	(“interview” OR “focus group” OR “focus group discussion” OR “group discussion” OR “in-depth interview” OR “semi-structured interview” OR “observation” OR “ethnography”) AND
E	Evaluation	(“perception” OR “experience” OR “belief” OR “attitude” OR “views” OR “knowledge” OR “practices” OR “healthcare seeking”) AND
R	Research type	(“qualitative” OR “mixed methods” OR “qualitative study” OR “qualitative research”)

Appendix Table 2. Characteristics of the included studies in qualitative evidence synthesis

No	Author (year)	Study setting	Aim	Study design	Sample size	Study participants	Data collection	Data analysis
1	Naserrudin, et al (2023)	Sabah, Malaysia	To highlight rural community members' perspectives regarding inequities to health relating to P. knowlesi malaria exposure.	Qualitative study	35	members of rural communities and community leaders	in-depth interviews & focus group discussions	reflexive thematic analysis
2	Naserrudin, et al (2023)	Sabah, Malaysia	To document local knowledge on malaria causation and preventive practices of rural communities in Sabah, Malaysia, using photovoice—a participatory research method.	Qualitative study	26	members of rural communities	focus group discussions	Thematic analysis
3	Naserrudin, et al (2023)	Sabah, Malaysia	To explore the barriers and facilitators related to prevention of mosquito bites among rural communities living in Sabah, Malaysia using the participatory visual method, photovoice.	Qualitative study	26	members of rural communities	focus group discussions	Thematic analysis
4	Kader Maideen et al. (2022)	central spine forest range in	To determine the sero-prevalence of malaria and the knowledge,	Mixed-methods study	13	Indigenous settlements	in-depth interviews	Thematic analysis

		Peninsular Malaysia	attitudes and practices relating to the prevention of malaria among the indigenous adults living in the central forest spine in Peninsular Malaysia.					
5	Azlan et al (2023)	Johor, Pahang, Kelantan (Malaysia)	To explores at-risk communities' attitudes toward Plasmodium knowlesi (P. knowlesi) malaria prevention behaviours using the Integrated Behavioural Model (IBM) as a guiding framework. This study also presents efforts and challenges faced by district health officers in Peninsular Malaysia in their role as the health authority for mitigating the disease.	Qualitative study	17	Local community, Indigenous settlement, and military personal	in-depth interviews	Thematic analysis

Appendix Table 3: Quality assessment of the included studies

ID	Author (Year)	Meta Domain				Method Domains- Research Design				Method Domains - Research Conduct				Overall
		Aim & Questions	Stakeholders	Researchers	Context	Research Strategy	Theory	Ethical Consideration	Equity, Diversity, and Inclusion	Participant Recruitment & Selection	Data Collection	Analysis & Interpretation	Presentation of Findings	
1	Naserrudin et al. (2023)	E	G	G	E	E	G	E	UC	G	G	G	E	Minimal
2	Naserrudin et al. (2023)	E	G	G	E	E	UC	E	G	G	E	G	E	Minimal
3	Naserrudin et al. (2023)	E	G	G	E	E	UC	E	G	G	E	G	E	Minimal
4	Maideen et al (2022)	E	G	F	E	E	UC	E	E	G	G	G	F	Minor
5	Azlan, et al (2023)	G	G	UC	E	G	UC	E	E	G	G	G	F	Minor

Quality assessment

E = Excellent, G = Good, F = Fair, P = Poor, UC = Unclear

Appendix Table 4: Themes Development of Community Experiences and Perceptions of *Plasmodium Knowlesi* Malaria, and Factors Influencing Prevention and Healthcare-Seeking Behaviors in Malaysia

**Codes**	**Subtheme**	**Descriptive Theme**	**Analytical Theme**
Awareness of malaria<br>Awareness of vector<br>Limited awareness of monkey malaria<br>Previous experience of research<br>Gain new knowledge<br>Self-motivation	Awareness of malaria and vector	Theme 1: Community Knowledge, Perception, and Experience of Knowlesi Malaria	Theme 1. Knowledge and Lived Realities Shape Community Risk Perception of Knowlesi Malaria
Fear of sickness and its socioeconomic burden<br>Fear of hospital admission<br>Risk for children<br>Perceived lucky<br>Perceived resistant	Perceived Severity and Risk		
Deforestation and natural habitat destruction<br>Seasonal variation in vector density<br>Vector breeding sites<br>Monkey population and human–wildlife conflict	Ecological and Environmental Challenges in Malaria Prevention	Theme 2: Barriers to Prevention and Healthcare-Seeking	Theme 2. Environmental, Structural, and Social Barriers Constrain Community Engagement with Malaria Prevention and Healthcare Seeking
Distance from healthcare<br>Limited provision of bed net<br>Transportation<br>No proper road<br>Electricity supply issues<br>Internet connection issues<br>Water supply issues	Structural and Access Barriers		
Family commitments<br>Fear of hospital-related costs<br>Housing conditions	Social and Household Barriers		

brick houses<br>Outdoor recreation, sports, and cultural practices			
Lack of trust in preventive measures<br>Challenge to use personal protection<br>Neglect of precautionary measures<br>Frustrations due to monkey and malaria<br>Cost of prevention (e.g., repellents, coils)<br>Worry about side effects of coils	Doubts on Effectiveness of Preventive Measures and Practical Limitations		
Use of garlic, plants, smoke, hand killing, coconut fiber<br>Smoke<br>Plants<br>Hand killing<br>Water collection and environmental cleaning<br>Self-prevention and household routines	Natural and Household Remedies	Theme 3: Local Prevention Strategies and Protective Practices	Theme 3. Malaria Prevention Practices Reflect Local Knowledge, and Availability of Formal Prevention Measures
Mosquito incense, coils, repellents<br>Prophylaxis medication<br>Bed nets	Modern and Formal Prevention Tools		
Needs: mosquito repellents, bed nets.<br>Malaria vaccine<br>Needs: effective vector control, monkey control	Tailored prevention measures for local context are needed		
